# Decreased plasma neuropeptides in first-episode schizophrenia, bipolar disorder, major depressive disorder: associations with clinical symptoms and cognitive function

**DOI:** 10.3389/fpsyt.2023.1180720

**Published:** 2023-05-19

**Authors:** Hua Yu, Peiyan Ni, Liansheng Zhao, Yang Tian, Mingli Li, Xiaojing Li, Wei Wei, Jinxue Wei, Wei Deng, Xiangdong Du, Qiang Wang, Wanjun Guo, Xiaohong Ma, Jeremy Coid, Tao Li

**Affiliations:** ^1^Department of Neurobiology, Affiliated Mental Health Center and Hangzhou Seventh People's Hospital, Zhejiang University School of Medicine, Hangzhou, Zhejiang, China; ^2^NHC and CAMS Key Laboratory of Medical Neurobiology, MOE Frontier Science Center for Brain Science and Brain-machine Integration, School of Brain Science and Brain Medicine, Zhejiang University, Hangzhou, Zhejiang, China; ^3^The Psychiatric Laboratory and Mental Health Center, West China Hospital, Sichuan University, Chengdu, Sichuan, China; ^4^Suzhou Psychiatry Hospital, Affiliated Guangji Hospital of Soochow University, Suzhou, Jiangsu, China

**Keywords:** schizophrenia, bipolar disorder, major depressive disorder, neuropeptide, disease monitoring

## Abstract

**Background:**

There is an urgent need to identify differentiating and disease-monitoring biomarkers of schizophrenia, bipolar disorders (BD), and major depressive disorders (MDD) to improve treatment and management.

**Methods:**

We recruited 54 first-episode schizophrenia (FES) patients, 52 BD patients, 35 MDD patients, and 54 healthy controls from inpatient and outpatient clinics. *α*-Melanocyte Stimulating Hormone (*α*-MSH), *β*-endorphin, neurotensin, orexin-A, oxytocin, and substance P were investigated using quantitative multiplex assay method. Psychotic symptoms were measured using the Brief Psychiatric Rating Scale (BPRS) and Positive and Negative Syndrome Scale (PANSS), manic symptoms using the Young Mania Rating Scale (YMRS), and depressive symptoms using 17 item-Hamilton Depression Rating Scale (HAMD). We additionally measured cognitive function by using a battery of tests given to all participants.

**Results:**

*α*-MSH, neurotensin, orexin-A, oxytocin, and substance P were decreased in the three patient groups compared with controls. Neurotensin outperformed all biomarkers in differentiating patient groups from controls. There were no significant differences for 6 neuropeptides in their ability to differentiate between the three patient groups. Higher neurotensin was associated with better executive function across the entire sample. Lower oxytocin and higher substance p were associated with more psychotic symptoms in FES and BD groups. *β*-endorphin was associated with early morning wakening symptom in all three patient groups.

**Conclusion:**

Our research shows decreased circulating neuropeptides have the potential to differentiate severe mental illnesses from controls. These neuropeptides are promising treatment targets for improving clinical symptoms and cognitive function in FES, BD, and MDD.

## Background

Schizophrenia (SCZ), bipolar disorder (BD), and major depressive disorders (MDD) are common and serious mental illnesses associated with substantial morbidity and mortality as well as high personal and societal costs (1). Although these disorders are a severe public health problem (2), our understanding and treatment of mental illnesses have lagged behind the progress of other medical fields (3). The main reason for this lag behind is the lack of understanding of the diseases’ etiopathology. Accumulating data from animal and human studies suggest that the pathophysiology of SCZ, BD and MDD are associated with monoamine abnormality (4–7). The monoamine hypothesis is supported by the clinical efficacy of many drugs for treating mental illness, however, there is usually a time lag between the acute pharmacological effects and clinical improvement (8). Furthermore, their therapeutic effects are limited, with high relapse rates, and a proportion of patients will deteriorate over the life course (9–11). Exploring the neuropathophysiological mechanisms of these disorders will strengthen our understanding of the disease etiology, which will further provide a basis for improving on effectiveness with improved side-effect profile compared to currently available therapies is of considerable importance (12, 13).

Recently, research into the mechanism and drug treatment of mental illness has focused increasingly on neuropeptides, which are distributed throughout the digestive, circulatory, and nervous systems, and can serve as neurotransmitters, neuromodulators, and hormones (11, 14, 15). Neuropeptides are often co-localized and co-released with monoamine neurotransmitters such as dopamine, glutamate, or γ-aminobutyric acid (GABA) (16), and can be detected peripherally. More than 100 neuropeptides have been identified, and most act *via* one or more of a correspondingly large number of 7-transmembrane, G protein-coupled receptors (GPCRs) (>200) (16). Neuropeptides and their receptors modulate many diverse functions of the central nervous system, including reward, sleep, emotion, and executive function (17–19). Previous studies have indicated *β*-endorphin, oxytocin, opioid peptides, orexin, and neurotensin (NT), are all implicated in mental illness (18, 20–22). Lower cerebral spinal fluid (CSF) neurotensin concentrations appear to be correlated with greater psychopathology severity, including thought disorder, deficit symptoms, disorganized behavior, and impaired functioning (14). NT deficiency impairs the working memory function (23), and NT genes variances were associated with executive function among healthy participants (19). Plasma oxytocin was reported to be correlated negatively with psychotic symptoms (24). Animal and experimental studies focused on neuropeptides in depression, found α-melanocyte stimulating hormone (MSH), and their receptors might have the potential to be treatment targets in stress-related mood disorders (25, 26). *β*-endorphin is the most important primary agonist of mu-opioid receptors (27). It is involved in reward-centric and homeostasis-restoring behaviors, which makes it a research target of interest in psychiatric disorders (27, 28).

Despite these findings, neuropeptide studies still offer little insight into either core pathophysiology or treatment options for SCZ, BD, and MDD (29). Precise brain neuropeptide measurement using CSF from lumbar punctures is invasive and not suitable for widespread use (30). In contrast, plasma detection of neuropeptides is less invasive, can accurately detect concentration, and be used widely in clinical research. However, how does the concentration of plasma neuropeptides change, and how does the plasma neuropeptides correlated with clinical symptom as well as cognitive function in these severe mental illnesses are being rarely explored. What’s more, whether these plasma neuropeptides had the potential to discriminate different disease status is still unknown. In this study, we used a new immuno-assay measurement to detect the plasma level of neuropeptides in first-episode schizophrenia (FES), BD, and MDD. Firstly, we explored whether these plasma neuropeptides showed the same change as it was reported in CSF in schizophrenia, BD and MDD, and whether the abnormal level of neuropeptides would be associated with clinical symptoms and cognitive function in patient groups. Finally, we tested whether these neuropeptides can be used as a biomarker to distinguish FES, BD and MDD from controls.

## Materials and methods

### Participants

FES, BD, MDD, and healthy control (HC) volunteers were recruited for the current study from in- and outpatient psychiatric facilities at West China Hospital of Sichuan University. We recruited 54 FES (26 male, 28 female), 52 BD (21 male, 31 female), and 35 MDD (15 male, 20 female), diagnosed according to standard operational criteria in the Diagnostic and Statistical Manual of Mental Disorders IV (DSM-IV). Fifty-four healthy volunteers (23 male, 31 female) were included. Healthy volunteers were screened for major psychiatric disorders using the Structured Clinical Interview for DSM-IV, non-patient edition. We used the Positive and Negative Symptom Rating Scale (PANSS), the Brief Psychiatric Rating Scale (BPRS), the Young Mania Rating Scale (YMRS), and 17-item Hamilton Depression Rating Scale (HAMD) to measure the symptom severity. The clinical rating scales are evaluated by experienced psychiatrists within 3 days after the patients are recruited in the study. Patients were excluded if they met the criteria for alcohol or substance abuse within 1 year of screening, and significant medical illness. All right-handed subjects provided written informed consent. The study was approved by the West China Hospital of Sichuan University Ethics committee, and the first subject was included in March 5th, 2015.

### Cognitive function measurement

Intelligence quotient (IQ), verbal IQ, and performance IQ scores of all participants were assessed using the seven-subtest short form of the revised Wechsler Adult Intelligence Scale in Chinese (31). The Cambridge Neuropsychological Test Automated Battery (CANTAB) is a computerized tool used to measure cognitive function in diverse populations (32). Stockings of Cambridge (SOC) is a part of the CANTAB task. Participants are shown two displays, each containing colored balls. Each participant must move the ball in the lower display to copy the pattern shown in the upper display. During the test, participants are asked to make as few moves as possible to match the two patterns. SOC problems solved in minimum moves is a fundamental measure, recording the number of occasions upon which the subject has completed a test problem in the minimum possible number of moves. It included mean moves for 2, 3, 4, and 5-move problems. Here, we used the problems solved in minimum moves for 5-move problems (MM5M). SOC-MM5M is a measure of spatial planning memory and executive function. A lower score indicates better performance.

### Plasma neuropeptide measurements

Not fasted blood samples were collected by venipuncture between 4.00 p.m. and 4.30 p.m. using ethylenediaminetetraacetic acid as an anti-coagulant. Peripheral blood mononuclear cells were removed by refrigerated centrifugation at 1,000 *g* for 10 min, and the separated plasma was immediately divided into 0.5-mL aliquots and stored at −80°C. Plasma factors were evaluated using MILLIPLEX® MAP kits (Merck KGaA, Darmstadt, Germany). The MILLIPLEX® MAP Human Neuropeptide Magnetic Bead Panel is used for the simultaneous quantification of the following 6 analytes in any combination: *α*-MSH, *β*-Endorphin, Neurotensin, Orexin-A, Oxytocin, and Substance P. This kit may be used for the analysis of all or any combination of the above analytes in tissue/cell lysate, culture supernatant samples, and CSF, serum or plasma samples. The immunoassay procedure was followed by the manufacture’s standard procedure and was described by our group elsewhere (15). Standard curves were generated using neuropeptide standards. Data were analyzed using a FLEXMAP 3D® instrument (Luminex, Merck Millipore) operated with xPONENT® software (version 4.0, Luminex). Median fluorescent intensity data were analyzed using a weighted 5-parameter logistic method to calculate the factor concentrations.

### Statistical analysis

Stata 14.0 and SPSS 24.0 were used for the analysis. The distribution of the continuous variables was checked using Shapiro–Wilk’s test. Log_10_ transformation was used to correct variables that were not normally distributed. Parametric comparisons (*t-*test or one-way ANOVA with post-hoc Bonferroni analysis). Fisher’s exact test was used to check the statistical significance of between-group differences. Spearman or Pearson correlation was conducted to test the correlation coefficient for categorical variables or continuous variables, respectively. The differences in cognitive function and the plasma neuropeptides among groups were tested by ANCOVA, with age, gender and BMI as covariates, the education year was additionally co-variated out for cognitive function analysis. The post-hoc group comparison was conducted with the alpha value set to 0.05 and with Bonferroni correction, which was a relatively strict method used to correct for multiple comparisons. The results are presented as mean ± standard deviation unless otherwise specified (Table 1). Differentiating performance of plasma markers was assessed using age-gender-BMI adjusted area-under-the-curve (AUC) values from receiver operating characteristic (ROC) analyses (FES vs. HC; BD vs. HC; MDD vs. HC; FES vs. BD; FEP vs. MDD; and BD vs. MDD). The test accuracy, sensitivity, specificity, predictive values and AUC (AUC: 0.9–1.0 = excellent; 0.8–0.9 = good; 0.7–0.8 = fair; 0.6–0.7 = poor; 0.5–0.6 = fail) were measured (33). Differences in AUCs were evaluated using bootstrapping (*n* = 1,000).

**Table 1:** Demographic and clinical characteristics of FES, BD, MDD and HC.

	FES M (SD) (*n* = 54)	BD M (SD) (*n* = 52)	MDD M (SD) (*n* = 35)	HC M (SD) (*n* = 54)	ANOVA/ANCOVA			
					*F*/*t*/*x*^2^	df	*P*, two-tail	*Post hoc* test
Age (Years)	22.02 (7.25)	28.92 (11.45)	30.26 (10.26)	27.94 (9.55)	6.95	3, 194	**<0.001**	FES < BD, MDD, HC
Education (years)	11.43 (2.98)	13.08 (3.23)	12.49 (4.15)	15.08 (3.59)	10.49	3, 194	**<0.001**	FES, BD, MDD < HC
BMI	20.67 (3.22)	23.39 (4.07)	21.10 (2.95)	20.95 (4.30)	5.82	3, 194	**<0.001**	BD > FES, MDD, HC
Gender (male /female)	26(M)/28(F)	21 (M)/31 (F)	15 (M)/20 (F)	23 (M)/31 (F)	0.70	3, 195	0.87	–
Smoking (yes/no)	7(yes)/34 (no)	8 (yes)/38(no)	7 (yes)/28 (no)	6 (yes)/45 (no)	1.73	3, 173	0.76	–
**Cognitive function (age, gender, education, BMI as covariates)**								
VIQ	96.47 (14.70)	107.10 (15.94)	105.10 (17.37)	112.48 (13.45)	7.63	3,166	**<0.001**	FES < BD, MDD, HC-
PIQ	84.13 (18.77)	95.35 (17.49)	100.72 (17.53)	108.35 (13.05)	10.52	3, 166	**<0.001**	FES < BD, MDD, HC
WAIS IQ	92.42 (14.93)	103.75 (16.40)	103.41 (17.50)	111.65 (13.34)	12.17	3, 166	**<0.001**	FES < BD, MDD, HC
Logical memory immediately	7.13 (4.62)	7.36 (4.56)	8.81 (5.06)	10.81 (4.24)	4.42	3, 178	**<0.01**	FES, BD < HC
Logical memory delayed	5.62 (3.90)	5.61 (3.93)	7.00 (4.42)	8.60 (4.34)	3.57	3, 175	**<0.05**	BD < HC
TMT-A-Time	40.35 (15.05)	44.32 (22.47)	34.68 (15.28)	37.18 (14.56)	2.84	3, 178	**<0.05**	–
TMT-B-Time	71.06 (32.03)	72.34 (39.29)	61.83 (45.68)	56.00 (25.32)	3.01	3, 178	**<0.05**	–
DSST	41.91 (14.39)	49.94 (18.04)	49.26 (18.69)	59.88 (19.80)	6.84	3, 175	**<0.001**	FES < BD, MDD, HC
SOC-MM5M	6.45 (3.14)	7.09 (2.63)	6.64 (2.33)	5.72 (2.12)	1.46	3, 170	0.23	–
**Plasma Neuropeptide (age, gender, BMI as covariates)**								
Log_10_ *α*-MSH	1.95 (0.22)	1.95 (0.30)	1.90 (0.25)	2.20 (0.24)	13.12	3, 177	**<0.001**	FES, BD, MDD < HC
Log_10_ *β*-Endorphin	2.63 (0.18)	2.58 (0.26)	2.62 (0.22)	2.69 (0.16)	3.02	3, 192	**<0.05**	BD < HC
Log_10_ Neurotensin	2.24 (0.14)	2.26 (0.17)	2.21 (0.13)	2.42 (0.12)	20.67	3, 192	**<0.001**	FES, BD, MDD < HC
Log_10_ Orexin A	2.78 (0.10)	2.81 (0.13)	2.78 (0.092)	2.88 (0.11)	7.68	3, 192	**<0.001**	FES, BD, MDD < HC
Log_10_ Oxytocin	2.28 (0.18)	2.37 (0.22)	2.24 (0.18)	2.49 (0.16)	15.19	3, 183	**<0.001**	FES, BD, MDD < HC
Log_10_ Substance P	1.51 (0.23)	1.56 (0.27)	1.46 (0.22)	1.75 (0.20)	14.39	3, 189	**<0.001**	FES, BD, MDD < HC
**Clinical parameters**								
HAMD total score	7.03 (5.12)	10.70 (8.03)	21.81 (5.50)	–	45.56	2, 112	**<0.001**	FES < BD < MDD
YMRS total score	5.00 (6.01)	10.07 (11.31)	–	–	−2.25	74	**0.027**	FES < BD
PANSS	86.24 (18.99)	54.09 (20.71)	–	–	7.64	87	**<0.001**	FES > BD
BPRS	46.70 (9.78)	32.30 (11.76)	–	–	2.39	87	**<0.001**	FES > BD
GAF	45.77 (12.80)	54.11 (13.63)	53.10 (10.05)	–	5.89	2, 122	**<0.01**	FES < BD, MD
Age first episode (years)	21.17 (7.44)	24.04 (10.03)	27.21 (11.20)	–	3.68	2, 120	**<0.05**	FES < MDD
Duration of illness (months)	12.79 (21.40)	65.45 (64.07)	39.21 (55.80)	–	11.03	2, 116	**<0.001**	FES < BD
Depressive episode	–	2.22 (1.48)	1.77 (1.07)	–	1.43	69	0.16	–
Manic/hypomanic episode	–	1.98 (1.64)	–	–	–	–	**–**	–
Antidepressants (Yes/No)	–	24/28	18/16	–	0.38	1, 86	0.54	–
Mood stabilizers (Yes/No)	–	32/20	–	–	–	–	**–**	–
Antipsychotics (Yes/No)	–	30/22	3/32		21.40	1, 87	**<0.001**	BD > MDD

FES, first episode schizophrenia; BD, bipolar disorder; MDD, major depressive disorder; HC, healthy controls; M, mean value; WAIS, Wechsler Adult Intelligence Scale Vocabulary Score (37 items); IQ, intelligence quotient; VIQ, verbal intelligence quotient; PIQ, performance intelligence quotient; TMT-A, Trail Making Test Parts A; TMT-B, Trail Making Test Parts B; DSST, digit symbol substitution test; SOC-MM5M, stockings of Cambridge, minimum move of 5 movement problems; MSH, melatonin stimulating hormone; HAMD, Hamilton depression rating scale (17 items); YMRS, Young Mania Rating Scale (11 items); PANSS, The Positive and Negative Syndrome Scale; BPRS, The Brief Psychiatric Rating Scale; GAF, Global Assessment of Functioning; n, number; SD, standard variance; M, male; F, female. For the comparison of neuropeptides, age, gender, and BMI were co-variated out. Significance level: *p* < 0.05, *p* < 0.01, *p* < 0.001. Bold represents statistically significant results (*p* < 0.05).

Finally, separate stepwise linear regression analyses were used to test associations between plasma neuropeptides and clinical symptom scores or cognitive function. The linear regression model used age, gender, education, BMI, group status, log_10_ α-MSH, log_10_ β-endorphins, log_10_ neurotensin, log_10_ orexin A, log_10_ oxytocin, and log_10_ substance P as independent variables. The least significant variables were removed one at a time until only significant variables remained. Significance was taken as *p* < 0.05. Supplementary Table S1 shows the factors excluded, respectively, in the linear regression models.

## Results

### Demographic and clinical characteristics

Demographic, clinical, plasma biomarkers, and cognitive features of patients and healthy controls are presented in Table 1. The FES group was younger than the other three groups. The BD group had a higher body mass index (BMI) compared to the other three groups. There were no gender differences between patients and controls. The three patient groups had lower educational levels than the controls. Compared to FES and BD, the MDD group showed significantly increased HAMD scores. Because few MDD subjects were measured with YMRS, PANSS, and BPRS, we only compared these symptom scores between FES and BD. The YMRS scores were significantly higher in the BD group compared to the FES group. The PANSS total score and BPRS total score were significantly increased in the FES group compared to the BD group. The BD group had a significantly longer illness duration than the FES group (see results in Table 1; Supplementary Table S2).

### Cognitive function analysis

ANCOVA analysis indicated that there were significant group differences in total IQ, and performance IQ, among the FES, BD, MDD, and HCs. *Post hoc* analyses revealed decreased total IQ scores, and performance IQ scores in the FES patient group compared to controls. There were no significant differences in SOC-MM5M scores between the patients and control groups (see Table 1).

### Different patterns of plasma neuropeptides in the four subject groups

**Figure 1:**
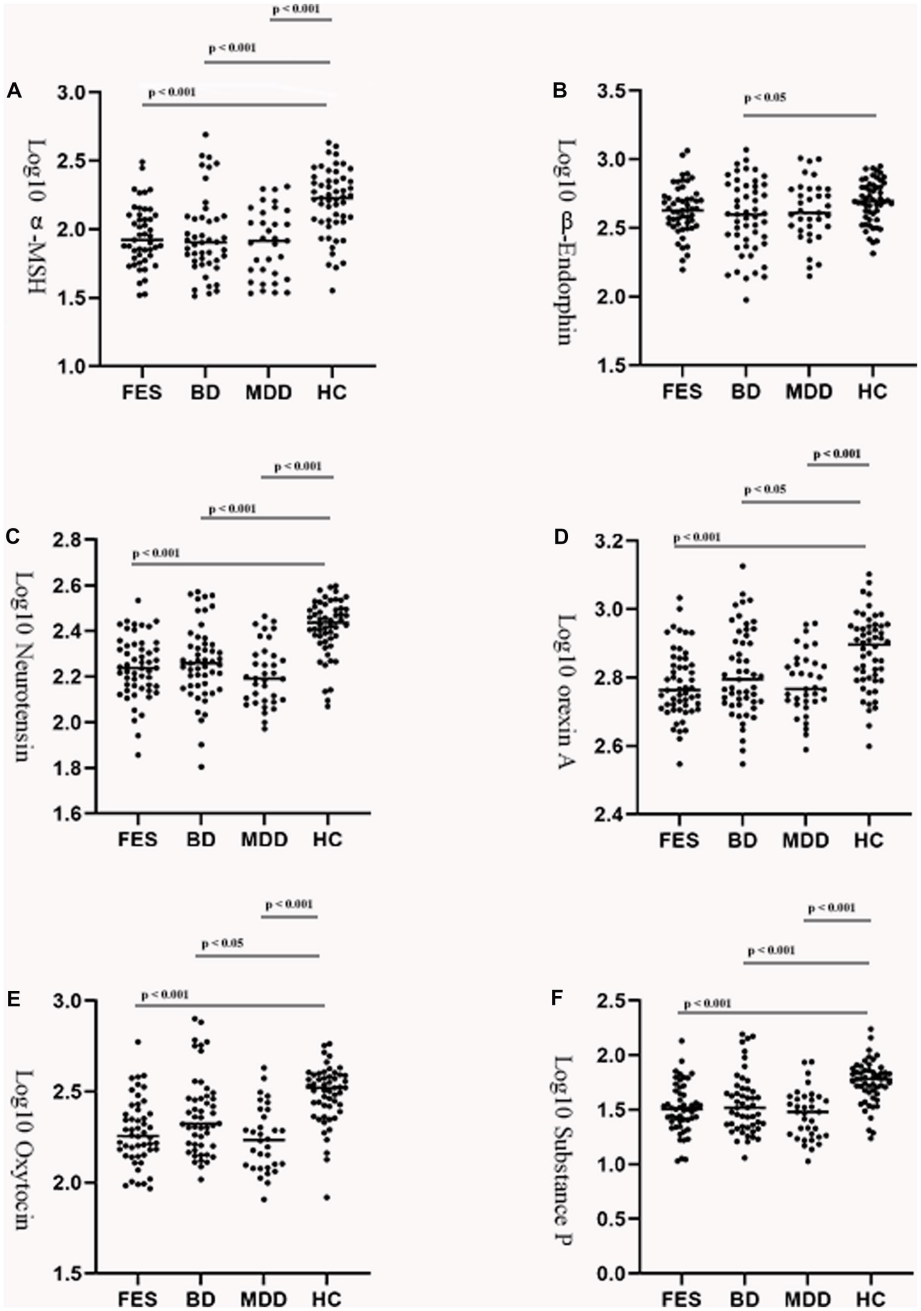
Group comparison of plasma neuropeptides among the patients and control group. Log transformed plasma *α*-melanocyte-stimulating hormone **(A)**, *β*-endorphin **(B)**, neurotensin **(C)**, orexin A **(D)**, oxytocin **(E)**, and substance P **(F)** levels in patients with FES, BD, or MDD and healthy controls. Horizontal lines in dotted plots denote mean values. MSH, melatonin stimulating hormone; FES, first episode schizophrenia; BD, bipolar disorders; MDD, major depressive disorders; HC, healthy controls.

Controlling for age, gender, and BMI, the log_10_ α-MSH, log_10_ neurotensin, log_10_ orexin A, log_10_ oxytocin, and log_10_ substance P level were significantly decreased in the three patient groups compared to controls. In addition, only the BD group showed decreased log_10_ *β*-endorphins compared to controls. We did not detect any significant differences between groups for six neuropeptides (see Table 1; Figure 1).

### Disease differentiating potential of plasma biomarkers

**Figure 2:**
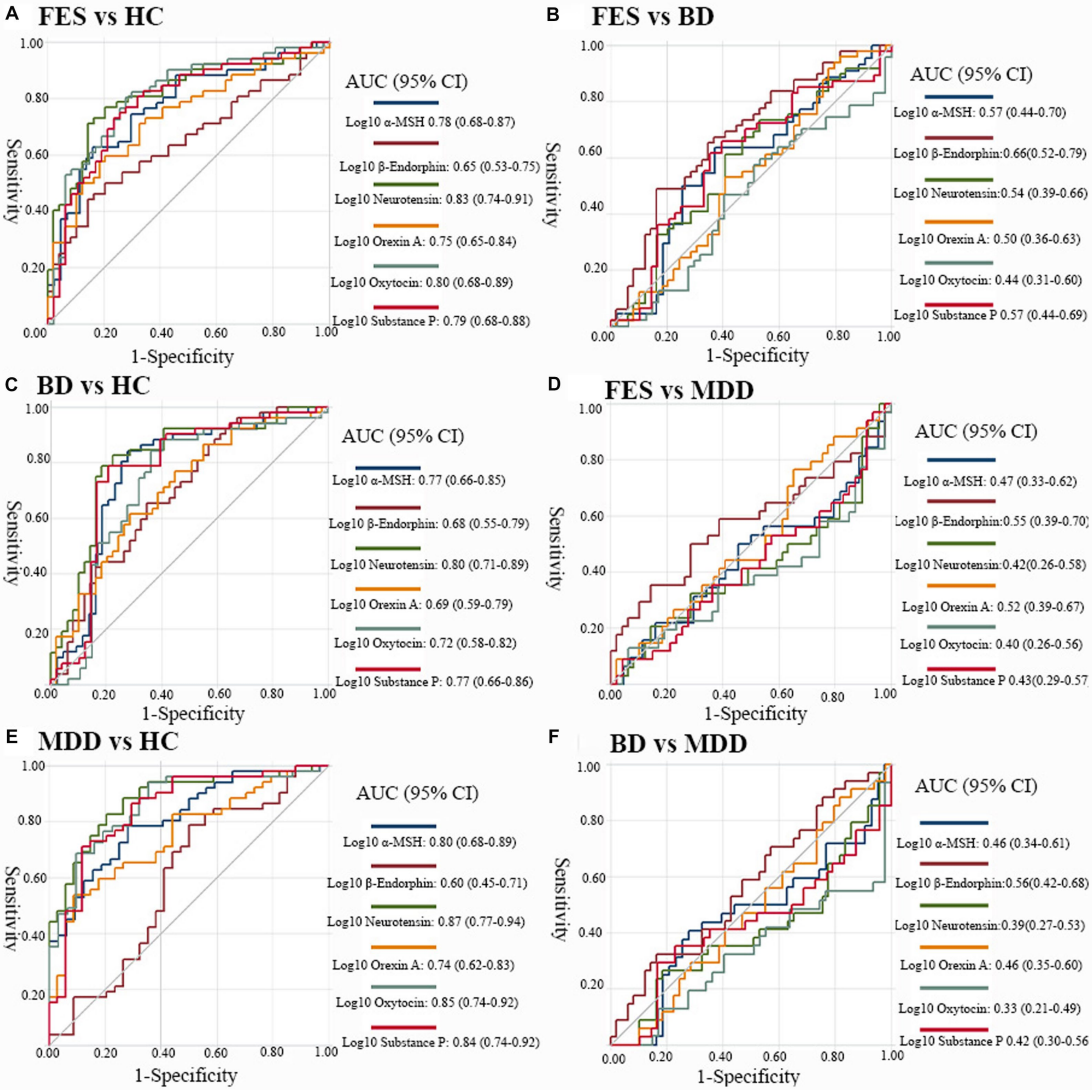
Discriminative performance of biomarkers across diagnostic groups. Receiver-operating characteristics (ROC) curves displaying the performance of plasma *α*-MSH, *β*-endorphins, neurotensin, orexin A, oxytocin and substance P to distinguish **(A)** FES from HCs, **(B)** BD from HCs, **(C)** MDD from HCs, **(D)** individuals with FES from BD, **(E)** FES versus MDD, and **(F)** BD versus MDD. MSH, melatonin stimulating hormone; FES, first episode schizophrenia; BD, bipolar disorders; MDD, major depressive disorders; HC, healthy controls; AUC, area under the curve. The AUCs were all covariated out age, gender and body mass index.

Receiver operating characteristic curves demonstrating the differentiating potential of plasma neuropeptides are shown in Figure 2. Across all comparisons, only patients compared with controls showed a good level of accuracy, and the differentiating performances between patient groups was poor. The highest AUC values were seen for plasma neurotensin when comparing patients with controls; FES versus HC [AUC = 0.83, 95% confidence interval [CI] 0.74–0.91; Figure 2A]; BD versus HC [AUC = 0.80, 95% CI 0.71–0.89; Figure 2B]; and MDD versus HC [AUC = 0.87, 95% CI 0.77–0.94; Figure 2C]. Other AUCs for discriminating patients from controls which showed good levels of discrimination above 0.80 included: oxytocin in FES vs. HC [AUC = 0.80, 95% CI 0.68–0.89; Figure 2C]; oxytocin in MDD vs. HC [AUC = 0.85, 95% CI 0.74–0.92; Figure 2C]; a-MSH in MDD vs. HC [AUC = 0.80, 95% CI 0.68–0.89; Figure 2C]; and substance P in MDD vs. HC [AUC = 0.84, 95% CI 0.74–0.92; Figure 2C]. For contrasts between patient groups, the AUCs are relatively poor (Figures 2D–F).

### Association between plasma neuropeptides and executive function

In the combined samples of the four groups, stepwise linear regression analysis using SOC-MM5M scores as the dependent variable and age, gender, education, BMI, group status, log_10_ *α*-MSH, log_10_ *β*-endorphins, log_10_ neurotensin, log_10_ orexin A, log_10_ oxytocin and log_10_ substance P as the independent variables showed that the linear regression model was significant (*F*_1,153_ = 8.01, *p* < 0.01). The adjusted multivariate coefficient of determination (*R*^2^) for the model was 0.05 for the predictor. The standardized *β* coefficient value for neurotensin was −0.22, with a *t* value of −2.83 (see Table 2; Supplementary Table S1).

**Table 2:** Association between neuropeptides with SOC-MM5M in the combined subject groups (step-wise regression).

	Unstandardized coefficients		Standardized coefficients	*t*	*p*	*R* ^2^	Adjusted *R*^2^	*F*
	*B*	SE	Beta					
Constant	15.47	3.25	–	4.76	<0.001	0.050	0.044	*F* (1, 153) = 8.01, *p* < 0.01
Log_10_ neurotensin	−3.97	1.40	−0.22	−2.83	<0.01			

Independent variable: age, gender, education, BMI, group status, log_10_ *α*-MSH, log_10_ *β*-endorphins, log_10_ neurotensin, log_10_ orexin A, log_10_ oxytocin and log_10_ substance P.

### Plasma neuropeptides were associated with psychotic symptoms in the FES and BD groups

The summary of linear regression analyses for evaluating the relationships among PANSS subscales and BPRS factor scores within FES and BD groups is presented in Table 3. When using age, gender, education, BMI, log_10_ α-MSH, log_10_ *β*-endorphins, log_10_ neurotensin, log_10_ orexin A, log_10_ oxytocin, and log_10_ substance P as the independent variables, and P2 (positive symptom scale item 2), N2 (negative symptom scale item 2), N3 (negative symptom scale item 3), and G9 (general pathology symptom scale item 9) as independent variables, we found that the regression models were all significant because all *p-*values were all lower than 0.05. Oxytocin was the only variable that could predict PANSS subscale symptom scores, with lower oxytocin levels predicting higher PANNS scores. When using the BPRS deficiency energy factor score as the dependent variable, we found that oxytocin level explained a significant amount of the variance in the BPRS deficiency energy factor score in Step 1 (*F* = 6.40, *p* < 0.05; *R*^2^ = 0.076; β = −0.28). In the second step, the inclusion of substance P (β = 4.33) enhanced the relationship between BPRS deficiency energy factor score and oxytocin level (*β* = −0.65) based on the magnitude of the standardized beta-coefficient in Step 2 (*F* = 5.36, *p* < 0.01; Δ*R*^2^ = 0.023). The adjusted multivariate coefficient of determination (*R*^2^) for the final model was 0.099 for the predictors. The standardized β coefficient value for oxytocin and substance P were − 0.65 and 4.33, with *t* values of −3.04 and 2.02 in step 2 (see results in Table 3 and Supplementary Table S1).

**Table 3:** Associations between neuropeptides with psychotic symptoms in FES and BD groups.

	Unstandardized coefficients		Standardized coefficients	*t*	*p*	*R* ^2^	Adjusted *R*^2^	*F*
	*B*	SE	*Beta*					
P2								
Constant	7.19	2.06	–	3.49	<0.01	0.062	0.050	*F*(1, 78) = 5.14, *p* < 0.05
Log_10_ oxytocin	−1.99	0.88	−0.25	−2.27	<0.05			
N2								
Constant	7.12	2.10	–	3.39	<0.01	0.054	0.042	*F*(1, 78) = 4.45, *p* < 0.05
Log_10_ oxytocin	−1.89	0.90	−0.23	−2.11	<0.05			
N3								
Constant	7.54	2.08		3.62	<0.01	0.061	0.049	*F*(1, 78) = 5.03, *p* < 0.05
Log_10_ oxytocin	−1.99	0.89	−0.25	−2.24	<0.05			
G9								
Constant	7.63	2.43	–	3.15	<0.01	0.049	0.037	*F*(1, 78) = 4.03, *p* < 0.05
Log_10_ oxytocin	−2.08	1.04	−0.22	−2.01	<0.05			
BPRS deficiency energy factor score								
Constant	24.47	5.90		4.15	<0.001			
Log_10_ oxytocin	−6.36	2.52	−0.28	−2.53	<0.05	0.076	0.064	*F*(1, 78) = 6.40, *p* < 0.05
Constant	32.01	6.89	–	4.65	<0.001	0.122	0.099	*F*(2, 77) = 5.36, *p* < 0.01
Log_10_ oxytocin	−15.03	5.00	−0.65	−3.04	< 0.01			
Log_10_ substance P	8.15	4.04	4.33	2.02	< 0.05			

Independent variables: age, gender, education years, BMI, log10 *α*-MSH, log10 *β*-endorphins, log10 neurotensin, log10 orexin A, log10 oxytocin and log10 substance P. P2, PANSS positive symptom scale item 2; N2, PANSS negative symptom scale item 2; N3, PANSS negative symptom scale item 3; G9, PANSS general pathology symptom scale item 9; PANSS, The Positive and Negative Syndrome Scale; BPRS, The Brief Psychiatric Rating scale.

### Plasma neuropeptide was associated with insomnia symptoms in all three psychiatric disorder groups

The summary of linear regression analyses for evaluating the relationships among depression severity, measured by the HAMD-17 item 6 (which is a measurement of early morning wakening), in the three patient groups is presented in Table 4. MDD group status explained a significant amount of the variance in early morning wakening severity in Step 1 (*F* = 41.88, *p* < 0.001; *R*^2^ = 0.30; *β* = 0.55). The inclusion of log_10_ endorphin (*β* = −0.25) enhanced the relationship between early morning awakening severity and MDD group status (*β* = 0.56) based on the magnitude of the standardized beta-coefficient in Step 2 (*F* = 27.69, *p* < 0.001; Δ*R*^2^ = 0.06). The adjusted multivariate coefficient of determination (*R*^2^) in step 2 was 0.36 for the predictors. The standardized *β* coefficient value for MDD group status and log_10_ endorphin were 0.56 and −0.25, with *t* values of 6.90 and −3.12 (see Table 4; Supplementary Table S1).

**Table 4:** Associations between neuropeptides and HAMD scores item 6 in the three patient groups.

	Unstandardized coefficients		Standardized coefficients	*t*	*p*	*R* ^2^	Adjusted *R*^2^	*F*
	*B*	SE	Beta					
Constant	0.38	0.077		4.92	<0.001	0.30	0.30	*F*(1, 96) = 41.88, *p* < 0.001
MDD group	0.95	0.15	0.55	6.47	<0.001			
Constant	2.91	0.81	–	3.57	<0.01	0.37	0.36	*F*(2, 95) = 27.69, *p* < 0.001
MDD group	0.97	0.14	0.56	6.90	<0.001			
Log_10_ endorphin	−0.96	0.31	−0.25	−3.12	<0.01			

Independent variables: age, gender, education, BMI, group status, log_10_ *α*-MSH, log_10_ *β*-endorphins, log_10_ neurotensin, log_10_ orexin A, log_10_ oxytocin and log_10_ substance P, HAMD: 17 item Hamilton depression rating scale. MDD, major depressive disorders.

### Partial correlation between plasma neuropeptides and other clinical information

We conducted correlation analyses between neuropeptides and illness duration, age of onset, mood episodes, and drug usage in the three patient groups, separately, but did not find any significant correlations in either FES or MDD group. However, in the BD group, we found plasma β-endorphin level was significantly positively correlated with illness duration (*r* = 0.41, *p* < 0.01) (see results in Supplementary Table S3).

## Discussion

In this study, we found that plasma α-MSH, orexin-A, oxytocin, neurotensin, and substance P, were all significantly decreased in FES, BD, and MDD groups compared to controls. *β*-endorphins were only decreased in the BD group compared to controls. In contrast, there were no significant differences observed among the three patient groups for six plasma neuropeptides. Neurotensin was the only plasma biomarker that could provide a relatively high differentiating potential to distinguish FES, BD, and MDD from controls (AUC = 0.83, 0.80, and 0.87 respectively). Furthermore, we have demonstrated that the plasma neuropeptides were differentially associated with specific clinical symptoms and executive function across disease groups. Our results indicate the disease monitoring and disease-categorizing potential of plasma neuropeptides, which could therefore be used in clinical and research settings (34).

Research evidence has already identified the role of neurotensin in the pathophysiology of psychosis and the mechanism of action of antipsychotic drugs (35, 36). Decreased CSF neurotensin concentration has been found in patients with schizophrenia, and improvements in overall psychopathology, including positive and negative symptoms, were correlated with increases in CSF neurotensin concentrations during treatment (14, 35). Our study is consistent with previous research and we find decreased neurotensin in first episode, never treated schizophrenia patients. There has been little previous investigation of the effects of neurotensin in BD and MDD. However, it was reported that neurotensin receptor 2 (NTSR2) was decreased in the anterior cingulate cortex of BD (37). NTSR2 mRNA and NTSR2 binding were reported to be down-regulated in transgenic mice expressing anxiety, stress, and depression (38). Furthermore, neurotensin receptor 1 (Ntsr1) knockout mice also showed increased anxiety and despair behaviors (39). Our results may be consistent with the hypothesis that plasma peptide changes are consistent across the major psychiatric disorders, as these disorders may have transdiagnostic properties (40). In our test of the differentiating performance of plasma neuropeptides, our finding that neurotensin has a good differentiating potential between patient groups and controls, with AUCs above 0.80, is new. Neurotensin is an endogenous tridecapeptide neurotransmitter that is heterogeneously distributed within the mammalian central nervous system (CNS) and has close neuroanatomical, functional associations with the dopamine neurotransmitter system (12, 41).

In our correlation analysis, we found that the measurement of SOC-MM5M was negatively associated with neurotensin. Our results are consistent with the previous study in humans which showed NT genes variances were associated with working memory performance among healthy participants (42). We expanded the association results to a larger spectrum of the human sample, which includes the FES, BD, MDD, and HCs. Evidence has indicated that neurotensin is often co-released with dopamine, and dopamine is widely expressed in the frontal lobe (43). As SOC-MM5M is a measurement of frontal lobe function (44), our results suggested that neurotensin might have the potential to influence frontal lobe function. However, further exploration is needed. Above all, our results support the possibility that plasma neurotensin has a central role in cognitive function, and it has the potential to be the treatment target for improving cognitive deficit in severe mental illness.

We found decreased oxytocin in plasma levels in schizophrenia as well as mood disorder patients. CSF and plasma oxytocin levels were found to be decreased in drug-naive schizophrenia patients and chronic schizophrenia patients and showed increased levels with antipsychotics drug treatment (45). Reduced plasma oxytocin concentrations were observed in patients with MDD and BD compared to controls (24, 46). Our results are consistent with previously published studies that reported decreased plasma oxytocin in schizophrenia, BD, and MDD (24, 45, 46). Furthermore, we found oxytocin was negatively correlated with negative and positive symptom subscales in bipolar and FES patients. Clinical studies have shown that nasal administration of oxytocin improves some symptoms of schizophrenia (47). Our results suggest that oxytocin plays a central role in the pathophysiology of major psychiatric illnesses, and increased oxytocin levels in FES and BD patients may help with the treatment of psychotic symptoms (8).

In our study, we only found decreased *β*-endorphin in the BD group compared with controls, and there were no differences between FES or MDD and controls. Few studies have explored endorphin levels in BD, although a single electroconvulsive therapy (ECT) study reported that after ECT treatment, the patient group had significantly increased endorphin levels (48). We found that a core symptom of depression, early-morning wakefulness of insomnia, was negatively associated with *β*-endorphin. Because insomnia is one of the most common symptoms of psychiatric illness, and a previous study has reported that electroacupuncture induced sleep enhancement may be mediated, in part, by increasing the concentrations of *β*-endorphin (49). Our finding is therefore consistent with *β*-endorphin having a role in the pathophysiology of insomnia symptoms in major psychiatric illnesses.

We also found decreased Orexin A, *α*-MSH, and Substance P across the three patient groups, and these three neuropeptides could discriminate patients from controls with modest accuracy (between 0.7 and 0.8). Loss of orexin neurons and decrease of orexin levels in plasma are observed in patients with depression, schizophrenia, and other neurodegenerative diseases (20, 50, 51). Our results may indicate that altered orexin-A signaling was disrupted in schizophrenia, BD, and MDD. *α*-MSH is one cleavage product of the pituitary hormone pro-opiomelanocortin (POMC), and numerous studies have described the role of POMC in metabolic syndrome (50). Psychopharmacotherapy strongly impacts the metabolic system, in particular in schizophrenia and bipolar disorders (52). As one of POMC’s downstream effector hormones, decreased α-MSH may be a potential risk factor for metabolic syndrome in several mental illnesses. We found that substance P was positively correlated with psychotic symptoms in FES and BD. Based on the fact that SP-containing neurons synapse with dopaminergic neurons in the midbrain, and that application of SP agonists in animal studies lead to increased dopaminergic turnover and locomotor activity (53–55), our results suggested that abnormal SP neurotransmission may be involved in the etiopathology of psychosis.

To our knowledge, there was only one study comparing the differences of the plasma neuropeptides between schizophrenia, BD and MDD subjects compared with controls (56). In Hidese et al’s (56) study, the authors reported there was no difference of plasma *α*-MSH, *β*-endorphin, neurotensin, oxytocin, and substance P between patient groups and controls in a larger sample size. And they did not find any significant correlation between these above-mentioned plasma neuropeptides with clinical symptom and cognitive function. The reasons for the quite different results might be that there were several differences in our study sample compared with theirs. First, most of their participants are taking psychotropic medication though they adjusted the medication effects in their analysis. However, in our research, we recruited first episode drug naïve schizophrenia patients as well as drug washed MDD subjects, and only BD patients in our research are taking drugs. Second, as they stated in their analysis that they did not record the somatic symptom in their subjects, as the interference of chronic somatic disorders such as diabetes, cardiovascular diseases, endocrinological problems, and their specific pharmacological treatment might have affected plasma neuropeptide levels. In our study, we excluded subjects with somatic symptom such as hypertension, endocrinological problems as well as patients taking any other pharmacological treatment. Finally, it was reported age has effect on the plasma proteins (57), and plasma neuropeptide concentration was found to increase significantly with age (58). Our patients have a relative younger mean age, as most of our subjects are at their 20th, while in Hidese et al’s (56) study, they recruited subjects at a mean age over 30th-40th. All these differences might contribute to the quite different research findings, further longitudinal study is needed.

There are several limitations that need to be addressed. The first limitation of the study is that it remains unclear whether plasma neuropeptide levels correlate with brain levels of neuropeptides because of the blood–brain barrier. In our study, we showed that plasma neuropeptides are correlated with cognitive function, psychotic and depressive symptoms. This may indicate that circulating neuropeptides can reflect the neuropeptide in the central nervous system (24). The second limitation is that drug treatment may confound our results, as BD and MDD groups were previously medicated. Although we also did not detect any significant correlation between drug usage and neuropeptide measurements, further studies must include first episode and drug naïve BD and MDD patients. Finally, the cross-sectional design of this study will not provide evidence of causality, and without longitudinal data, it is not possible to establish a true cause and effect relationship (59). We, therefore suggest that longitudinal observation of changes of neuropeptides in severe mental illness should be conducted in future research.

## Conclusion

In conclusion, this study explored the plasma concentration of six neuropeptides, including α-MSH, β-Endorphin, Neurotensin, Orexin-A, Oxytocin, and Substance P in FES, BD, and MDD, and tested their differentiating potential to distinguish patients from controls. We also found there were significant correlation between plasma neuropeptides and psychotic, depressive symptoms, as well as executive function in FES, BD, and MDD groups. If our results are confirmed in further large-scale longitudinal studies, we could conclude plasma neuropeptides might be promising targets for treating clinical symptoms and cognitive deficits.

## Data availability statement

The raw data supporting the conclusions of this article will be made available by the authors, without undue reservation.

## Ethics statement

The studies involving human participants were reviewed and approved by West China Hospital of Sichuan University Ethics committee. The patients/participants provided their written informed consent to participate in this study.

## Author contributions

HY, PN, JC, and TL developed the study, had full access to all data, and took responsibility for data integrity and accuracy. HY, PN, QW, and WG drafted the manuscript. WW, JW, XD, WD, and XM collected the data. YT, LZ, ML, and XL performed all data analyses. All authors agree to be accountable for all aspects of the work, ensuring that questions related to the accuracy or integrity of the data and results are appropriately investigated and resolved, and critically revised and approved the final version of the manuscript.

## Funding

This work was supported by the National Natural Science Foundation of China Key Project (81630030 and 81920108018 to TL), National Natural Science Foundation of China (81871054 and 81501159 to PN, 82101598 to HY), Special Foundation for Brain Research from Science and Technology Program of Guangdong (2018B030334001 to TL), 2021 Project for Hangzhou Medical Disciplines of Excellence and Key Project for Hangzhou Medical Disciplines, 1.3.5 Project for Disciplines of Excellence at West China Hospital of Sichuan University (ZY2016103, ZY2016203, and ZYGD20004 to TL), and Introductory Project of the Suzhou Clinical Expert Team (SZYJTD201715 to XD and TL).

## Conflict of interest

The authors declare that the research was conducted in the absence of any commercial or financial relationships that could be construed as a potential conflict of interest.

## Publisher’s note

All claims expressed in this article are solely those of the authors and do not necessarily represent those of their affiliated organizations, or those of the publisher, the editors and the reviewers. Any product that may be evaluated in this article, or claim that may be made by its manufacturer, is not guaranteed or endorsed by the publisher.

## Abbreviations

SCZ: schizophrenia
FES: first-episode schizophrenia
BD: bipolar disorder
MDD: major depressive disorder
DSM: Statistical Manual of Mental Disorders
GABA: γ-aminobutyric acid
GPCRs: G protein-coupled receptors
CSF: cerebral spinal fluid
NT: neurotensin
CSF: cerebral spinal fluid
NT: neuotensin
BPRS: Brief Psychiatric Rating Scale
YMRS: Young Mania Rating Scale
HAMD-17: 17-item Hamilton Depression Rating Scale
IQ: Intelligence quotient
CANTAB: Cambridge Neuropsychological Test Automated Battery
SOC: Stockings of Cambridge
MM5M: minimum moves for 5-move problems
AUC: area under the curve
ROC: values from receiver operating characteristic
BMI: body mass index
AIC: Akaike information criterion
SP: substance p
CI: confidence interval
NTSR1: neurotensin receptor 1 gene
MSH: melanocyte-stimulating hormone
