# Supplementary material for: Decreased plasma neuropeptides in first-episode schizophrenia, bipolar disorder, major depressive disorder: associations with clinical symptoms and cognitive function
Source: Front Psychiatry. 2023 May 19;14:1180720. doi: 10.3389/fpsyt.2023.1180720 (PMC10235770; doi:10.3389/fpsyt.2023.1180720)
Supplement: Supplementary file 1 [file Data_Sheet_1.docx]

**Supplementary Table 1. Factors excluded in step-wise linear regression.**

|  | **β** | **t value** | **P value** | **Partial correlation** | **Collinearity statistics tolerance** |
| --- | --- | --- | --- | --- | --- |
| **SOC-MM5M** | | | | | |
| MDD group status | 0.00 | 0.0050 | 1.00 | 0.00 | 0.93 |
| BD group status | 0.12 | 1.50 | 0.14 | 0.12 | 0.99 |
| FES group status | -0.074 | -0.93 | 0.35 | -0.075 | 0.97 |
| HC group status | -0.055 | -0.61 | 0.54 | -0.050 | 0.76 |
| Log_10_ MSH | -0.082 | -0.57 | 0.58 | -0.047 | 0.31 |
| Log_10_ Endorphin | 0.048 | 0.53 | 0.60 | 0.043 | 0.75 |
| Log_10_ Orexin | 0.087 | 0.77 | 0.44 | 0.062 | 0.49 |
| Log_10_ Oxytocin | 0.13 | 0.91 | 0.36 | 0.074 | 0.30 |
| Log_10_ Substance P | 0.20 | 1.35 | 0.18 | 0.11 | 0.30 |
| Gender | -0.011 | -0.14 | 0.89 | -0.011 | 1.00 |
| Age | 0.11 | 1.38 | 0.17 | 0.11 | 0.99 |
| Education years | -0.077 | -0.97 | 0.33 | -0.079 | 0.99 |
| BMI | 0.12 | 1.58 | 0.12 | 0.13 | 1.00 |
| **P2** | | | | | |
| Log_10_ MSH | 0.12 | 0.69 | 0.49 | 0.079 | 0.41 |
| Log_10_ Endorphin | 0.23 | 1.67 | 0.099 | 0.19 | 0.62 |
| Log_10_ Neurotensin | 0.056 | 0.28 | 0.78 | 0.032 | 0.31 |
| Log_10_ Orexin A | 0.070 | 0.40 | 0.69 | 0.046 | 0.40 |
| Log_10_ Substance P | 0.34 | 1.57 | 0.12 | 0.18 | 0.25 |
| Gender | 0.030 | 0.27 | 0.79 | 0.031 | 1.00 |
| Age | -0.019 | -0.16 | 0.87 | -0.018 | 0.88 |
| Education years | -0.19 | -1.71 | 0.091 | -0.19 | 1.00 |
| BMI | -0.034 | -0.30 | 0.77 | -0.034 | 0.97 |
| **N2** | | | | | |
| Log_10_ MSH | 0.20 | 1.13 | 0.26 | 0.13 | 0.41 |
| Log_10_ Endorphin | 0.23 | 1.66 | 0.10 | 0.19 | 0.62 |
| Log_10_ Neurotensin | 0.28 | 1.41 | 0.16 | 0.16 | 0.31 |
| Log_10_ Orexin | -0.0080 | -0.044 | 0.97 | -0.0050 | 0.40 |
| Log_10_ Substance P | -0.045 | -0.20 | 0.84 | -0.023 | 0.25 |
| Gender | 0.076 | 0.69 | 0.49 | 0.078 | 1.00 |
| Age | -0.14 | -1.21 | 0.23 | -0.14 | 0.88 |
| Education years | -0.080 | -0.72 | 0.47 | -0.082 | 1.00 |
| BMI | -0.056 | -0.50 | 0.62 | -0.056 | 0.97 |
| **N3** | | | | | |
| Log_10_ MSH | 0.31 | 1.82 | 0.073 | 0.20 | 0.41 |
| Log_10_ Endorphin | 0.21 | 1.54 | 0.13 | 0.17 | 0.62 |
| Log_10_ Neurotensin | 0.16 | 0.81 | 0.42 | 0.092 | 0.31 |
| Log_10_ Orexin A | -0.0020 | -0.014 | 0.99 | -0.0020 | 0.40 |
| Log_10_ Substance P | 0.099 | 0.45 | 0.66 | 0.051 | 0.25 |
| Gender | 0.062 | 0.56 | 0.58 | 0.064 | 1.00 |
| Age | -0.18 | -1.57 | 0.12 | -0.18 | 0.88 |
| Education years | -0.16 | -1.43 | 0.16 | -0.16 | 1.00 |
| BMI | -0.060 | -0.54 | 0.59 | -0.061 | 0.97 |
| **G9** | | | | | |
| Log_10_ MSH | 0.20 | 1.12 | 0.25 | 0.13 | 0.41 |
| Log_10_ Endorphin | 0.063 | 0.45 | 0.66 | 0.051 | 0.62 |
| Log_10_ Neurotensin | 0.28 | 1.40 | 0.17 | 0.16 | 0.31 |
| Log_10_ Orexin A | 0.16 | 0.90 | 0.37 | 0.10 | 0.40 |
| Log_10_ Substance P | 0.35 | 1.60 | 0.12 | 0.18 | 0.25 |
| Gender | -0.053 | -0.48 | 0.63 | -0.055 | 1.00 |
| Age | -0.091 | -0.77 | 0.44 | -0.088 | 0.88 |
| Education years | -0.058 | -0.52 | 0.60 | -0.059 | 1.00 |
| BMI | -0.17 | -1.52 | 0.13 | -0.17 | 0.97 |
| **BPRS deficiency energy factor score** | | | | | |
| Log_10_ MSH | 0.055 | 0.31 | 0.76 | 0.035 | 0.35 |
| Log_10_ Endorphin | 0.081 | 0.58 | 0.57 | 0.066 | 0.58 |
| Log_10_ Neurotensin | -0.036 | 0.17 | 0.87 | -0.019 | 0.26 |
| Log_10_ Orexin A | -0.089 | -0.46 | 0.64 | -0.053 | 0.32 |
| Gender | 0.011 | 0.10 | 0.92 | 0.011 | 0.98 |
| Age | -0.12 | -1.03 | 0.31 | -0.12 | 0.87 |
| Education years | -0.13 | -1.16 | 0.25 | -0.13 | 0.95 |
| BMI | -0.19 | -1.77 | 0.081 | -0.20 | 0.97 |
| **HAMD-6** | | | | | |
| Log_10_ MSH | -0.021 | -0.21 | 0.84 | -0.022 | 0.65 |
| Log_10_ Neurotensin | -0.033 | -0.33 | 0.74 | -0.034 | 0.68 |
| Log_10_ Orexin | -0.028 | -0.29 | -.78 | -0.030 | 0.72 |
| Log_10_ Oxytocin | 0.044 | 0.41 | 0.69 | 0.042 | 0.58 |
| Log_10_ Substance P | -0.039 | -0.36 | 0.72 | -0.038 | 0.60 |
| Gender | 0.019 | 0.22 | 0.82 | 0.023 | 1.00 |
| Age | 0.14 | 1.71 | 0.091 | 0.17 | 0.94 |
| Education years | 0.022 | 0.26 | 0.80 | 0.027 | 0.98 |
| BMI | -0.083 | 0.99 | 0.33 | -0.10 | 0.96 |
| BD group status | 0.095 | 1.00 | 0.32 | 0.10 | 0.74 |
| FES group status | -0.091 | 1.00 | 0.32 | -0.10 | 0.82 |

Abbreviations: MDD: major depressive disorder; BD: bipolar disorder; FES: first episode schizophrenia; P2: PANSS positive symptom scale item 2; N2: PANSS negative symptom scale item 2; N3: PANSS negative symptom scale item 3; G9: PANSS general pathology symptom scale item 9; PANSS: The Positive and Negative Syndrome Scale; BPRS: The Brief Psychiatric Rating Scale.

**Supplementary Table 2. Group comparisons of clinical symptom sub-scales among FES, BD, and MDD groups.**

|  | **FES M (SD)**  **(n=54)** | **BD M (SD)**  **(n=52)** | **MDD M (SD)**  **(n=35)** | **HC M (SD)**  **(n=54)** | **ANOVA/ANCOVA** | | | |
| --- | --- | --- | --- | --- | --- | --- | --- | --- |
|  |  |  |  |  | **F / x²** | **df** | **P, 2-tail** | **Post hoc test** |
| **Clinical parameters** | | | | | | | | |
| **HAMD total score** | 7.03 (5.12) | 10.70 (8.03) | 21.81 (5.50) | - | 45.56 | 2, 109 | **< 0.001** | FES <BD < MDD |
| **HAMD-1** | 1.09 (0.96) | 1.35 (1.40) | 3.06 (0.84) | - | 29.93 | 2, 109 | **< 0.001** | FES, BD < MDD |
| **HAMD-2** | 0.15 (0.44) | 0.54 (0.75) | 1.38 (0.98) | - | 23.10 | 2, 109 | **< 0.001** | FES, BD < MDD |
| **HAMD-3** | 0.12 (0.48) | 0.57 (0.98) | 1.34 (1.04) | - | 16.45 | 2, 109 | **< 0.001** | FES, BD < MDD |
| **HAMD-4** | 0.29 (0.52) | 0.96 (0.84) | 1.50 (0.80) | - | 21.57 | 2, 109 | **< 0.001** | FES, BD < MDD |
| **HAMD-5** | 0.38 (0.55) | 0.70 (0.73) | 1.44 (0.76) | - | 20.49 | 2, 109 | **< 0.001** | FES, BD < MDD |
| **HAMD-6** | 0.26 (0.51) | 0.48 (0.69) | 1.34 (0.70) | - | 26.21 | 2, 109 | **< 0.001** | FES, BD < MDD |
| **HAMD-7** | 1.38 (1.52) | 1.46 (1.59) | 2.59 (0.95) | - | 7.86 | 2, 109 | **< 0.001** | FES, BD < MDD |
| **HAMD-8** | 0.59 (0.70) | 0.39 (0.65) | 1.13 (0.49) | - | 13.30 | 2, 109 | **< 0.001** | FES, BD < MDD |
| **HAMD-9** | 0.71 (0.84) | 0.87 (0.89) | 0.88 (1.01) | - | 0.40 | 2, 109 | 0.67 | - |
| **HAMD-10** | 0.26 (0.67) | 0.59 (0.78) | 1.28 (0.77) | - | 16.13 | 2, 109 | **< 0.001** | FES, BD < MDD |
| **HAMD-11** | 0.18 (0.46) | 0.50 (0.75) | 1.19 (0.90) | - | 16.74 | 2, 109 | **< 0.001** | FES, BD < MDD |
| **HAMD-12** | 0.18 (0.46) | 0.33 (0.56) | 0.94 (0.72) | - | 16.02 | 2, 109 | **< 0.001** | FES, BD < MDD |
| **HAMD-13** | 0.24 (0.50) | 0.35 (0.53) | 0.88 (0.75) | - | 11.18 | 2, 109 | **< 0.001** | FES, BD < MDD |
| **HAMD-14** | 0.09 (0.29) | 0.30 (0.59) | 0.66 (0.60) | - | 9.94 | 2, 109 | **< 0.001** | FES, BD < MDD |
| **HAMD-15** | 0.06 (0.24) | 0.30 (0.59) | 0.69 (0.74) | - | 10.44 | 2, 109 | **< 0.001** | FES, BD < MDD |
| **HAMD-16** | 0.18 (0.58) | 0.54 (0.86) | 1.28 (0.89) | - | 16.54 | 2, 109 | **< 0.001** | FES, BD < MDD |
| **HAMD-17** | 0.88 (0.98) | 0.48 (0.84) | 0.28 (0.46) | - | 45.56 | 2, 109 | **< 0.001** | FES > MDD |
| **YMRS total score** | 5.00 (6.01) | 10.07 (11.31) | - | - | -2.25 | 74 | **<0.05** | FES < BD |
| **YMRS-1** | 0.00 (0.00) | 1.04 (1.30) | - | - | -4.39 | 74 | **< 0.001** | FES < BD |
| **YMRS-2** | 0.07 (0.37) | 1.11 (1.34) | - | - | -4.16 | 74 | **< 0.001** | FES < BD |
| **YMRS-3** | 0.03 (0.18) | 0.39 (0.83) | - | - | -2.32 | 74 | **0.023** | FES < BD |
| **YMRS-4** | 0.07 (0.37) | 0.80 (1.05) | - | - | -3.71 | 74 | **< 0.001** | FES < BD |
| **YMRS-5** | 1.07 (1.60) | 1.24 (1.65) | - | - | -0.45 | 74 | 0.65 | - |
| **YMRS-6** | 0.20 (0.76） | 1.65 (2.25) | - | - | -3.40 | 74 | **< 0.01** | FES < BD |
| **YMRS-7** | 0.40 (0.72) | 0.61 (0.86) | - | - | -1.10 | 74 | 0.27 | - |
| **YMRS-8** | 1.37 (2.78) | 1.54 (2.18) | - | - | -0.31 | 74 | 0.76 | - |
| **YMRS-9** | 0.40 (0.86) | 0.33 (0.76) | - | - | 0.39 | 74 | 0.70 | - |
| **YMRS-10** | 0.20 (0.55) | 0.54 (1.03) | - | - | -1.68 | 74 | 0.097 | - |
| **YMRS-11** | 1.20 (1.65) | 0.80 (1.33) | - | - | 1.15 | 74 | 0.25 | - |
| **PANSS** | 86.24 (18.99) | 54.09 (20.71) | - | - | 7.64 | 87 | **< 0.001** | FES > BD |
| **PANSS-PS-1** | 4.76 (1.20) | 1.84 (1.41) | - | - | 10.56 | 87 | **< 0.001** | FES > BD |
| **PANSS-PS-2** | 3.37 (1.51) | 1.70 (1.26) | - | - | 5.64 | 87 | **< 0.001** | FES > BD |
| **PANSS-PS-3** | 3.80 (1.59) | 1.40 (0.88) | - | - | 8.78 | 87 | **< 0.001** | FES > BD |
| **PANSS-PS-4** | 1.98 (1.51) | 2.49 (1.70) | - | - | -1.50 | 87 | 0.14 | - |
| **PANSS-PS-5** | 1.39 (0.93) | 2.09 (1.54) | - | - | -2.62 | 87 | 0.10 | - |
| **PANSS-PS-6** | 4.35 (1.46) | 1.67 (1.23) | - | - | 9.30 | 87 | **< 0.001** | FES > BD |
| **PANSS-PS-7** | 2.98 (1.53) | 1.72 (1.26) | - | - | 4.22 | 87 | **< 0.001** | FES > BD |
| **PANSS-NS-1** | 3.48 (1.39) | 1.70 (1.12) | - | - | 6.60 | 87 | **< 0.001** | FES > BD |
| **PANSS--NS-2** | 3.63 (1.34) | 1.65 (1.13) | - | - | 7.51 | 87 | **< 0.001** | FES > BD |
| **PANSS-NS-3** | 3.78 (1.26) | 1.95 (1.33) | - | - | 6.66 | 87 | **< 0.001** | FES > BD |
| **PANSS-NS-4** | 3.83 (1.40) | 1.91 (1.44) | - | - | 6.36 | 87 | **< 0.001** | FES > BD |
| **PANSS-NS-5** | 2.78 (1.47) | 1.47 (1.12) | - | - | 4.72 | 87 | **< 0.001** | FES > BD |
| **PANSS-NS-6** | 3.37 (1.40) | 1.53 (1.05) | - | - | 6.93 | 87 | **< 0.001** | FES > BD |
| **PANSS-NS-7** | 2.43 (1.44) | 1.37 (0.93) | - | - | 4.11 | 87 | **< 0.001** | FES > BD |
| **PANSS-GP-1** | 1.85 (1.14) | 1.95 (1.15) | - | - | -0.44 | 87 | 0.66 | - |
| **PANSS-GP-2** | 2.13 (1.09) | 2.30 (1.23) | - | - | -0.70 | 87 | 0.49 | - |
| **PANSS-GP-3** | 1.33 (0.79) | 1.37 (0.87) | - | - | -0.26 | 87 | 0.80 | - |
| **PANSS-GP-4** | 2.57 (1.34) | 1.86 (1.08) | - | - | 2.71 | 87 | **< 0.01** | FES > BD |
| **PANSS-GP-5** | 1.28 (0.72) | 1.16 (0.49) | - | - | 0.92 | 87 | 0.36 | - |
| **PANSS-GP-6** | 2.67 (1.27) | 3.07 (1.93) | - | - | 0.92 | 87 | 0.36 | - |
| **PANSS-GP-7** | 2.48 (1.35) | 1.70 (1.15) | - | - | 2.94 | 87 | **< 0.01** | FES > BD |
| **PANSS-GP-8** | 2.09 (1.36) | 1.42 (0.98) | - | - | 2.64 | 87 | 0.10 | - |
| **PANSS-GP-9** | 3.85 (1.71) | 1.63 (1.16) | - | - | 7.12 | 87 | **< 0.001** | FES > BD |
| **PANSS-GP-10** | 1.33 (0.60) | 1.12 (0.39) | - | - | 1.94 | 87 | 0.055 | - |
| **PANSS-GP-11** | 3.67 (1.67) | 3.28 (1.53) | - | - | 1.16 | 87 | 0.25 | - |
| **PANSS-GP-12** | 4.22 (1.76) | 2.14 (1.46) | - | - | 6.04 | 87 | **< 0.001** | FES > BD |
| **PANSS-GP-13** | 3.17 (1.45) | 1.77 (0.10) | - | - | 5.22 | 87 | **< 0.001** | FES > BD |
| **PANSS-GP-14** | 2.37 (1.62) | 2.05 (1.34) | - | - | 1.02 | 87 | 0.31 | - |
| **PANSS-GP-15** | 2.09 (1.50) | 1.12 (0.39) | - | - | 4.11 | 87 | **< 0.001** | FES > BD |
| **PANSS-GP-16** | 3.22 (1.59) | 1.67 (1.19) | - | - | 5.15 | 87 | **< 0.001** | FES > BD |
| **PANSS-S1** | 2.35 (1.51) | 1.56 (1.16) | - | - | 2.75 | 87 | **< 0.01** | FES > BD |
| **PANSS-S2** | 2.02 (1.26) | 1.51 (0.94) | - | - | 2.16 | 87 | **< 0.05** | FES > BD |
| **PANSS-S3** | 3.26 (1.34) | 2.37 (1.50) | - | - | 2.94 | 87 | **< 0.01** | FES > BD |
| **BPRS total** | 46.70 (9.78) | 32.30 (11.76) | - | - | 2.39 | 87 | **< 0.001** | FES > BD |
| **BPRS Anxde** | 7.98 (3.17) | 8.70 (3.83) | - | - | -0.97 | 87 | 0.34 | - |
| **BPRS act** | 11.07 (3.65) | 6.47 (3.32) | - | - | 6.20 | 87 | **< 0.001** | FES > BD |
| **BPRS ang** | 12.41 (3.40) | 6.81 (3.92) | - | - | 7.21 | 87 | **< 0.001** | FES > BD |
| **BPRS susp** | 5.83 (2.24) | 5.51 (2.44) | - | - | 0.63 | 87 | 0.53 | - |
| **BPRS td** | 9.41 (3.07) | 4.81 (3.17) | - | - | 6.95 | 87 | **< 0.001** | FES > BD |

1.FES: first episode schizophrenia; BD: bipolar disorder; MDD: major depressive disorder; HC: healthy controls; HAMD: Hamilton depression rating scale (17 items); YMRS: Young Mania Rating Scale (11 items); BPRS: Brief Psychiatric Rating Scale (1); BPRS td: BPRS anxde: BPRS anxiety-depression factor; BPRS act: BPRS activation; BPRS ang: BPRS deficiency energy factor score/lacking in activity factor or withdrawal factor; BPRS susp: BPRS hostile-suspiciousness; BRPS td: BPRS thinking disturbance factor.

2.Significance level: p<0.05, p<0.01, p<0.001. Bold represents statistically significant results (p<0.05)

**Supplementary Table 3. Correlation analysis between neuropeptides and other clinical information.**

|  | **Log_10_ MSH** | **Log_10_ β-Endorphin** | **Log_10_ Neurotensin** | **Log_10_ Orexin A** | **Log_10_ Oxytocin** | **Log_10_ Substance P** |
| --- | --- | --- | --- | --- | --- | --- |
|  | *r (p)* | *r (p)* | *r (p)* | *r (p)* | *r (p)* | *r (p)* |
| **FES** | | | | | | |
| Illness duration | 0.30 (0.068) | -0.24 (0.15) | -0.22 (0.19) | -0.11 (0.52) | 0.038 (0.83) | -0.064 (0.71) |
| Age Onset | -0.13 (0.46) | -0.16 (0.33) | 0.15 (0.35) | 0.090 (0.58) | 0.088 (0.60) | 0.013 (0.94) |
| **BD** | | | | | | |
| Illness duration | -0.11 (0.52) | **0.41 (0.0060)**** | 0.037 (0.81) | 0.13 (0.40) | 0.071 (0.66) | 0.10 (0.52) |
| Age onset | 0.29 (0.075) | 0.19 (0.22) | 0.28 (0.064) | 0.13 (0.41) | 0.26 (0.094) | **0.31 (0.041)*** |
| Depressive Episodes | -0.049 (0.78) | 0.11 (0.50) | 0.0090 (0.96) | -0.092 (0.57) | 0.19 (0.26) | 0.055 (0.74) |
| Mani/hypomanic episode | -0.16 (0.35) | -0.12 (0.48) | -0.067 (0.68) | -0.071 (0.66) | -0.078 (0.64) | -0.16 (0.34) |
| Antidepressants | 0.065 (0.67) | 0.20 (0.17) | 0.16 (0.26) | -0.040 (0.78) | 0.20 (0.18) | 0.21 (0.15) |
| Mood stabilizers | -0.080 (0.60) | 0.030 (0.83) | 0.012 （0.93） | 0.082 (0.57） | -0.095 (0.52) | -0.18 (0.20) |
| Antipsychotics | 0.13 (0.39) | -0.030 (0.84) | -0.017 (0.90) | 0.19 (0.17) | 0.046 (0.75) | -0.0080 (0.95) |
| **MDD** | | | | | | |
| Illness duration | 0.16 (0.39) | 0.18 (0.32) | -0.0050 (0.98) | 0.20 (0.25) | -0.055 (0.77) | 0.072 (0.69) |
| Age onset | -0.017 (0.93) | -0.0090 (0.93) | -0.18 (0.96) | -0.038 (0.83) | 0.20 (0.29) | -0.021 (0.91) |
| Depressive Episodes | -0.13 (0.52) | -0.057 (0.77) | -0.19 (0.32) | -0.039 (0.84) | -0.24 (0.23) | -0.11 (0.27) |
| Antidepressants | 0.20 (0.27) | 0.018 (0.92) | 0.096 (0.59) | -0.19 (0.28) | 0.043 (0.82) | -0.030 (0.87) |
| Antipsychotics | -0.089 (0.62) | -0.061 (0.73) | -0.16 (0.35) | -0.10 (0.56) | -0.17 (0.36) | -0.11 (0.53) |

1. Abbreviations: MSH: melatonin stimulating hormone; FES: first episode psychosis; BD: bipolar disorders; MDD: major depressive disorders.

2. Significance level: *p < 0.05, **p < 0.01, ***p < 0.001.

**References**

1. CRIPPA JA, SANCHES RF, HALLAK JE, LOUREIRO SR, ZUARDI AW. Factor structure of Bech's version of the Brief Psychiatric Rating Scale in Brazilian patients. Brazilian journal of medical and biological research = Revista brasileira de pesquisas medicas e biologicas. 2002 Oct;35:1209-13.

2. AKAIKE H. Information Theory and an Extension of the Maximum Likelihood Principle. In: Parzen E, Tanabe K, Kitagawa G, eds. Selected Papers of Hirotugu Akaike. New York, NY: Springer New York; 1998. p. 199-213.
